# Impact of the brainstem in normal and impaired human movement control

**DOI:** 10.1113/JP290565

**Published:** 2026-09-09

**Authors:** Volker Dietz, Linard Filli

**Affiliations:** ^1^ Spinal Cord Injury Center Balgrist University Hospital, University of Zürich Zurich Switzerland; ^2^ Swiss Paraplegic Research Nottwil Switzerland

**Keywords:** brainstem function, cooperative hand movements, corticospinal tract, human motor control, locomotion, movement performance, recovery of mobility, reticulospinal tract

## Abstract

The corticospinal tract represents a highly developed neural system that dominates motor control in humans. This allows the performance of skilled finger movements, such as playing the piano. In contrast, according to preclinical studies, automatically performed complex movements, such as locomotion or cooperative forelimb movements, appear to be controlled mainly by brainstem motor centres. Only in recent years, with the advancement of new methodologies, has evidence emerged that limb coordination in humans is also based on neural limb coupling mediated by brainstem motor centres. This neural limb coupling is reflected in the observation that, during a movement, unilateral electrical nerve stimulation elicits muscle responses not only in the stimulated but also in the contralateral limb(s), involved in the respective motor task. The finding that activation of the reticulospinal system by loud acoustic stimuli evokes muscle responses that closely resemble the configuration of ‘coupling responses’ induced by electrical nerve stimulation suggests a contribution of the brainstem to the neural coupling mechanism. In addition to its role in interlimb coordination during stepping or cooperative hand movements, the reticulospinal system seems to be involved in the control of other motor tasks, including posture, reaching and force development. Furthermore, recent evidence indicates that brainstem motor centres contribute to functional recovery after stroke and spinal cord injury. These observations suggest that the brainstem plays a major role in motor control not only in animals but also in humans.

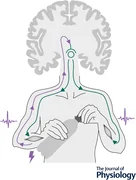

## Graphical abstract

**Abstract figure legend** Opening a bottle by neural limb coupling. When opening a bottle, the force exerted by the opening hand automatically matches the force exerted by the holding hand. This neural limb coupling is experimentally reflected by the observation that unilateral nerve stimulation (here: distal ulnar nerve, right forearm) during the opening movement elicits EMG responses in forearm muscles of both sides. This mechanism is suggested to be under the control of the brainstem, presumably via the reticulospinal system.

## Basic aspects of brainstem motor function

In vertebrates, the corticospinal (CS) and reticulospinal (RS) pathways are the two key descending motor systems (Tapia et al., 2022). Little is known about their relative contributions to movement control. It is assumed that their involvement varies depending on the motor task being performed. The CS system represents the dominant motor pathway in humans and is known to facilitate and control skilled movements of the hands and fingers (Lemon, 2019). In non‐human primates, disruption of the CS tract induces paresis of the fingers and toes, while basic motor behaviours such as standing, walking and climbing movements are only little impaired (Gilman & Marco, 1971; Lawrence & Kuypers, 1968; Zaaimi et al., 2012). The fact that CS control is primarily focused on fine‐tuned, dexterous movements of distal muscles implies that important aspects of human motor control are mediated by other motor systems, with brainstem nuclei being likely candidates.

While the CS system can be explored and modulated by various methods, the function of brainstem systems is more difficult to assess non‐invasively in humans. Consequently, little is known about the role of brainstem nuclei, specifically the RS system, in human motor control. The RS system is phylogenetically highly conserved and consists of an assembly of various nuclei distributed throughout the pons and medulla. Neurophysiological findings in animals indicate that RS fibres project diffusely to inter‐ and motoneurons of both sides of the spinal cord (Davidson & Buford, 2006; Riddle et al., 2009) and that the RS pathway is capable of translating a unilateral cortical command signal to both sides of the body, thereby contributing to movement coordination (Brocard et al., 2010).

There is growing neuroanatomical and neurophysiological evidence that the RS system plays a key role in motor control in vertebrates. For example, RS involvement has been described in the control of posture and locomotion (Drew et al., 2004), as well as for upper limb function (Riddle et al., 2009). This is consistent with the observation that the medullary part of the reticular formation plays an essential role in the motor control of the upper limbs in both animals (Esposito et al., 2014; Falasconi et al., 2025; Zhang et al., 2024) and humans (Jindal et al., 2026). More recent experiments using chemo‐ and optogenetic tools have confirmed the crucial involvement of the RS system in the control of locomotion (Hagglund et al., 2010; Juvin et al., 2016) and reaching movements in rodents (Ruder et al., 2021). Furthermore, brainstem motor nuclei are thought to integrate and process proprioceptive information during stepping in mice (Santuz et al., 2022).

This ‘Topical Review’ aims to highlight and discuss the current knowledge regarding the involvement of brainstem centres, particularly the RS system, in the coordination and control of stereotyped functional movements, such as opening a bottle or stepping, in humans. In recent years, novel techniques have been developed to activate the RS system and to investigate its role in human motor physiology. These include the application of loud acoustic stimuli (Carlsen et al., 2007; Valls‐Sole et al., 1995) and the analysis of ipsilateral motor‐evoked potentials in response to transcranial magnetic stimulation (TMS) (Mooney et al., 2025). These methodological approaches enable the exploration of the role of the RS in human motor control. Recent studies in primates and humans indicate an involvement of the RS system in force control (Glover & Baker, 2022) and the facilitation of explosive movements (Eilfort & Filli, 2025). RS drive appears to be particularly strong to upper limb flexors, including those contributing to power grasp tasks (Baker & Perez, 2017), and to lower limb extensors (Eilfort et al., 2025). Furthermore, indirect evidence suggests an impact of the RS system in movement coordination based on neural limb coupling (Dietz et al., 2024).

This neural limb coupling underlying movement coordination is believed to be mediated by the RS system on the basis of the following observations from preclinical and clinical experiments. First, electrical microstimulation of RS neurons evokes bilateral muscle responses in cats and primates, reflecting the divergent RS projections to inter‐ and motoneurons on both sides of the spinal cord (Drew & Rossignol, 1990a,b; Davidson & Buford, 2004, 2006). Second, unilateral motor commands from the cortex are distributed to both sides of the body via the RS system (Brocard et al., 2010). Third, in humans, the RS drive to acoustic stimuli is greater during bimanual than unimanual limb movements (Maslovat et al., 2020).

In conclusion, the CS tract has undergone considerable evolutionary development and has become the dominant motor system in primates. However, accumulating evidence over the last few years indicates that the RS system plays an essential role in basic aspects of motor control, not only in vertebrates but also in humans. Indeed, recent research suggests that the RS system is essentially involved in basic aspects of motor control across vertebrate species, and this appears to be the case in humans as well. The RS system contributes to the coordination of automatically performed, complex limb movements, such as locomotion, through the mechanism of neural interlimb coupling.

## Brainstem involvement in human movement control

Skilled uni‐ or bilateral precision finger movements, such as playing the piano, are known to be controlled by the CS system through direct cortico‐motoneuronal connections. The CS pathway is considered the dominant motor system in humans (Caldelari et al., 2020; Lemon, 2008). In contrast, recent studies indicate that everyday functional upper and lower limb movements, such as opening a bottle (Dietz et al., 2015; Thomas et al., 2018), or stepping (Dietz et al., 2001), are performed automatically with only limited influence of conscious (i.e. cortical) control.

### Limb coordination by neural coupling

Current studies provide evidence that the execution and coordination of complex movements are based on a ‘neural coupling’ of the upper and/or lower limbs (Dietz et al., 2001). This neural coupling is reflected in the observation that unilateral, non‐noxious nerve stimulation elicits EMG responses not only in muscles of the stimulated limb but also in contralateral limb muscles involved in the respective motor task. For example, during locomotion, unilateral distal tibial nerve stimulation evokes EMG responses in leg and proximal arm muscles on both sides (Dietz et al., 2001). This distribution of muscle responses reflects a neural linkage of cervical and thoraco‐lumbar propriospinal circuits that coordinate arm and leg movements of both sides during locomotion. Accordingly, upper limb swing during stepping appears to be an integral part of locomotor activity, reflecting the persistence of a quadrupedal organization in bipedal gait (Dietz, 2002). This mechanism is suggested to be under the control of the brainstem, presumably the RS system.

A specific walking condition is obstacle‐avoidance stepping. As soon as an obstacle is recognized, that is, prior to crossing the obstacle, larger response amplitudes to unilateral tibial nerve stimulation appear in arm and leg muscles on both sides (Dietz et al., 2001). This finding suggests that movement control is mediated by a combined contribution of the RS and CS systems.

**Figure 1:**
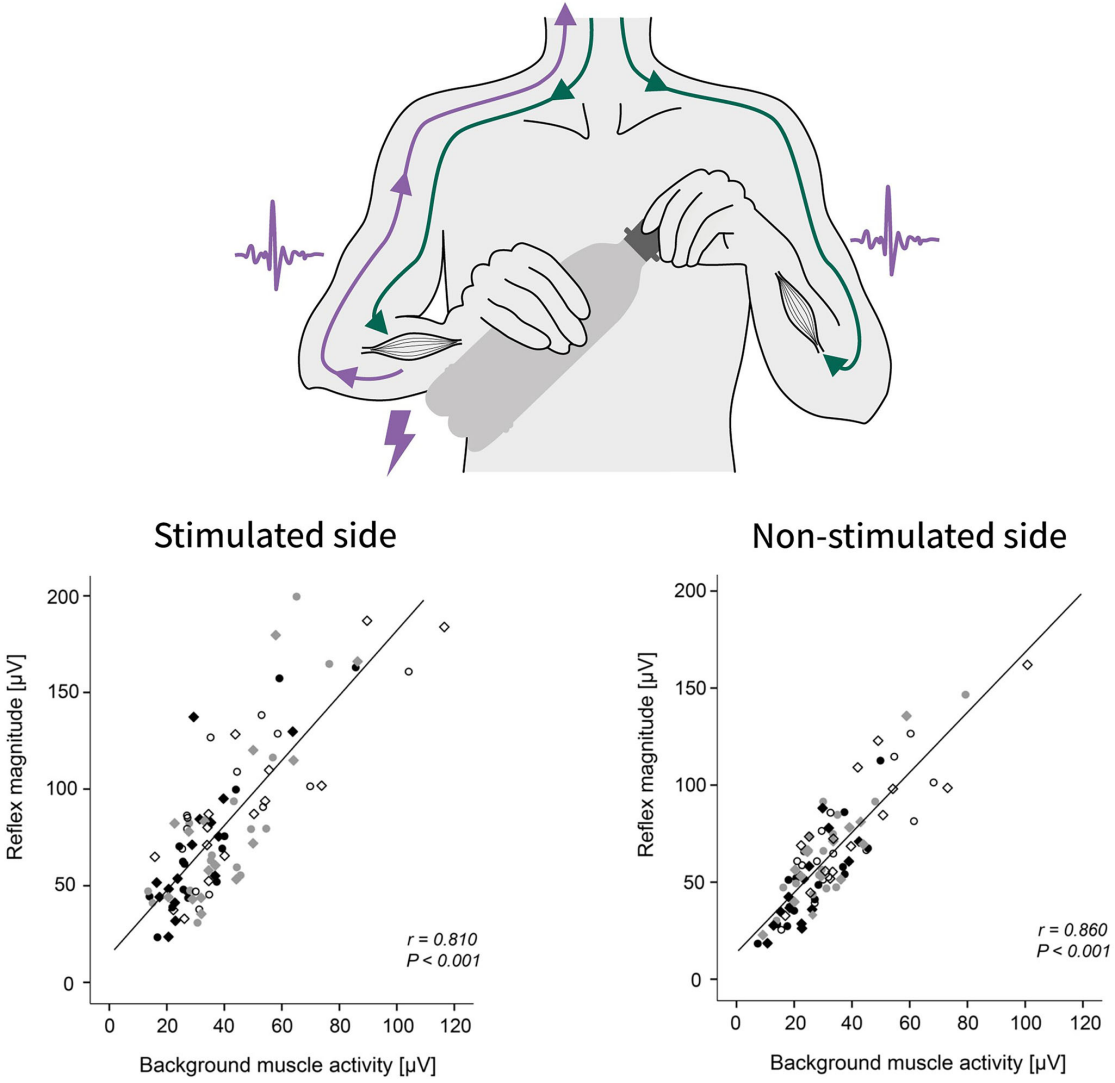
Schematic illustration of the control of cooperative hand movements through neural limb coupling The ‘opening a bottle’ task was performed at different movement velocities and force levels by the two hands, leading to different levels of background EMG activity on both sides. A linear relationship was observed between the level of background EMG activity (arising from the different movement velocities and applied forces) and the reflex response amplitudes on both the stimulated and the non‐stimulated sides. The two graphs shown below are adapted from Thomas et al. (2018).

Corresponding to neural limb coupling during locomotion, this system also contributes to movement coordination in other motor tasks. For example, neural limb coupling is involved in cooperative upper limb movements, such as opening a bottle (Dietz et al., 2015). Again, unilateral stimulation of a distal arm nerve evokes EMG responses in the forearm muscles on both sides, with amplitudes depending on the level of background muscle activity (automatic reflex gain scaling; Matthews, 1986). In this condition, the background EMG activity (which is at the same level on both sides) reflects the automatic matching of the force and movement velocity exerted by the opening hand with the force and velocity exerted by the opposite, holding hand. This occurs with the goal: two hands, one action (Thomas et al., 2018; Fig. 1). The close similarity of the EMG responses on the stimulated and non‐stimulated sides suggests that they are generated by a common neural structure.

It should be noted that neural upper limb coupling occurs not only during cooperative but also during non‐functional bilateral hand movements (Dietz et al., 2024).

Several candidates within the central nervous system (CNS) might be responsible for mediating the neural limb coupling mechanism. For example, the coupling could be based on the activation of both the ipsi‐ and contralateral cortices, connected via the corpus callosum (Carson et al., 2004), or alternatively through the non‐crossing corticospinal tract (Morecraft et al., 2013). However, according to recent studies, both mechanisms appear unlikely to be involved in neural limb coupling (Dietz et al., 2024; Morecraft et al., 2013). The reasons for this assumption are, first, that the ipsilateral CS tract seems to be functionally not relevant (Maitland & Baker, 2021) and, second, that neural coupling is partially preserved in individuals with stroke (Schrafl‐Altermatt & Dietz, 2016). These observations make the involvement of both hemispheres in neural coupling rather unlikely. Based on findings in non‐human primates, commissural interneurons in the cervical spinal cord might contribute to interlimb coordination (Soteropoulos et al. 2013)

### Mediation of neural limb coupling by the RS system

Only in recent years has evidence emerged that, corresponding to observations made in animal experiments, the RS pathway might play a major role in neural interlimb coordination through a neural coupling mechanism. There is growing evidence that the RS system represents an essential motor structure underlying the execution of automatic, complex movements, while precise movement control is mediated by the CS or by an interaction between the CS and RS pathways (Zaaimi et al., 2018).

For decades, techniques have been available to activate the CS system and analyse its role in motor control in humans. In contrast, only in recent years has it become feasible to activate and explore the RS system by analysing EMG responses in moving limb muscles evoked by loud tones or TMS (Fisher et al., 2012; Mooney et al., 2023). The application of loud acoustic stimuli (LAS) can considerably shorten reaction times, an effect that has been attributed to activation of the RS system (the so‐called ‘StartReact effect’; Tapia et al. 2022).

Besides the StartReact effect, LAS can also elicit other brainstem‐mediated responses through activation of the RS system. This approach was recently used to investigate a potential involvement of the RS system in neural coupling of the upper limbs (Dietz et al., 2024). LAS, combined with unilateral electrical stimulation (ES) of the ulnar nerve, were delivered either randomly, or in close succession during bilateral hand movements. EMG responses to LAS closely resembled those induced by ES in their configuration; that is, both response types showed highly similar negative‐to‐positive peak characteristics, response durations and peak latencies.

The fact that LAS‐evoked responses are known to activate neurons within the reticular formation provides indirect evidence that the responses to both stimuli might be mediated by the RS system. Consequently, the RS system appears to be involved in limb coordination through neural coupling during automatically performed motor tasks, such as cooperative hand movements or stepping (Dietz et al., 2024). However, a contribution of cortical, alternative subcortical or spinal systems to the neural coupling cannot be excluded. In the case of obstacle‐avoidance stepping, which requires transient conscious and visual control of movement, an interaction and cooperation between the RS and CS pathways is assumed to occur (Darling et al., 2018; Fisher et al., 2021; Haefeli et al., 2011). Furthermore, findings from experimental CS lesions indicate that certain aspects of corticomotor control are mediated through the cortico‐reticulospinal (CRS) system, and that the CRS pathway can compensate for some impaired CS function (Asboth et al., 2018; Lawrence & Kuypers, 1968). Therefore, the CRS system is considered to represent an important component of the motor control network across vertebrate species.

In early infancy, movement control is characterized by a fixed, automatic neural limb coupling due to an immature CS tract. This is reflected by the involuntary co‐activation of the contralateral limb during unilateral arm or hand movements (Soska et al., 2012). Only with the maturation of the CS system during late infancy does the subject gradually acquire the ability to perform uni‐ and bilateral precision movements of the hands and fingers (Koerte et al., 2011). Correspondingly, older individuals with impaired CS tract function, for example following a stroke, show a higher incidence of involuntary co‐activation of the contralateral limb (i.e. mirror movements). This limb co‐activation is suggested to be associated with an upregulated RS system (Ejaz et al., 2018).

**Figure 2:**
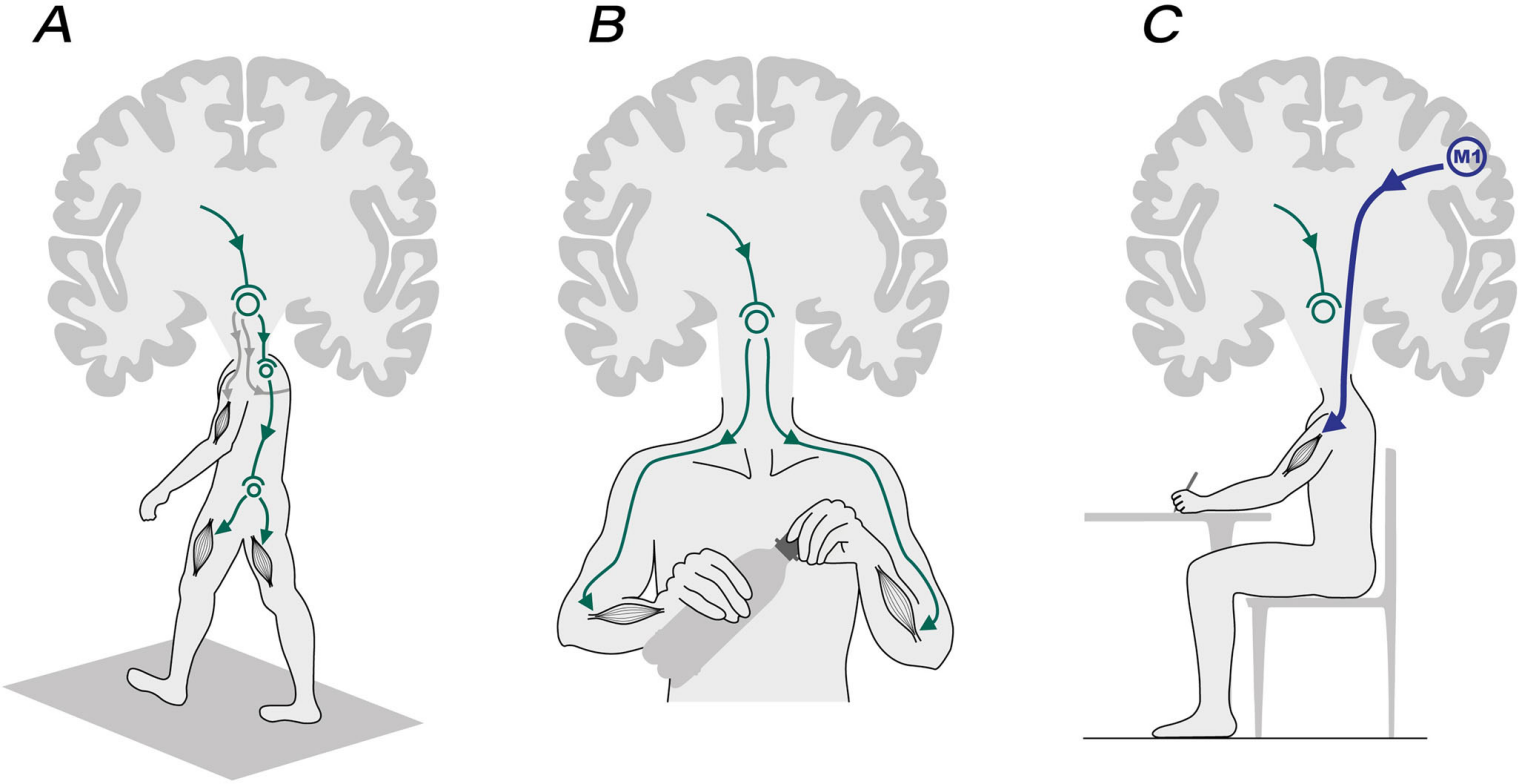
Schematic illustration of human motor control under three different movement conditions *A*, during locomotion, an unspecific impulse (e.g. from the periphery or the cortex) to brainstem nuclei leads to RS drive to cervical and thoracolumbar neural circuits, activating the upper and lower limbs on both sides for stepping movements. Arm swing is assumed to reflect a functional component of the quadrupedal organization underlying bipedal gait. This organization is suggested to be under the control of brainstem structures. *B*, during the task of ‘opening a bottle’, a neural coupling between the hands on both sides takes place. The force exerted by the opening hand is automatically matched to that exerted by the holding hand. This neural coupling of the hands is suggested to be under the control of the RS system; that is, a unilateral impulse to brainstem neurons leads to a bilateral executive drive. *C*, skilled hand and finger movements, such as writing or playing the piano, are assumed to be controlled predominantly by the cortico‐motoneuronal system, although RS projections are also known to contribute to fine finger movements (Soteropoulos et al., 2012).

In conclusion, indirect evidence suggests that stereotyped, complex everyday movements are controlled predominantly by the RS system (Fig. 2*A* and *B*; Table 1). In particular, this system is assumed to mediate neural coupling for limb coordination during automatically performed movements. This neural limb coupling seems to be robust, emerging even during non‐functional hand movements (Dietz et al. 2024). This raises the question of why neural limb coupling does not interfere with the performance of tasks in which the two hands must move independently, such as playing the violin? One possible explanation is that such bilateral hand movements are learned and extensively trained early in life, leading to punctual suppression of neural limb coupling.

**Table 1:** Evidence for a brainstem involvement in human interlimb coordination

1. Mediation of neural limb coupling: Unilateral nerve stimulation of a limb during a complex movement task leads not only to ipsilateral muscle responses but also to responses in contralateral muscles of the cooperating limb.
2. *Activation of the RS system by loud tones during movement*: Muscle responses to loud acoustic stimuli, which are known to activate the RS system, exhibit response characteristics that closely resemble EMG responses elicited by electrical nerve stimulation.
3. *Muscle responses to ES of the unaffected limb in stroke subjects*: ‘Normal’ responses appear in muscles of the contralateral, paretic limb.
4. *Bilateral distribution of unilateral motor commands by brainstem structures*: Animal experiments have shown that brainstem structures can translate unilateral inputs to the RS system into bilateral motor outputs, reflecting neural limb coupling. Consequently, the brainstem is assumed to play a major role in motor control not only in animal but also in human.

In primates, RS‐mediated movement control is supplemented by the CS system, which enables skilled, fractionated finger movements (Fig. 2*C*). In many movement tasks, the two systems interact and work in parallel to achieve accurate movement performance. The reticular formation is influenced by cortico‐reticular connections, which provide the brainstem with motor‐related information (Darling et al., 2018; Fisher et al., 2021).

## Brainstem involvement in the recovery of function after CNS damage

Preclinical findings imply that RS neurons possess a substantial capacity for neuroplastic adaptations and appear to play a crucial role in functional recovery after stroke (Bachmann et al., 2014; Choudhury et al., 2019; Herbert et al., 2015; Taga et al., 2024) and spinal cord injury (SCI) (Asboth et al., 2018; Filli et al., 2014; Zorner et al., 2014). These observations are consistent with neurophysiological findings in non‐human primates. Following an experimental lesion of the CS tract, the RS system contributes substantially to the recovery of impaired complex movements, including climbing and walking (Zaaimi et al., 2012).

Little is known about the role of brainstem motor centres in the recovery of movement function following stroke or SCI in humans. Damage to the CS pathway is known to be associated with poor recovery of individual finger function. Following a stroke, recovery of finger function reaches only about 20% (Rowe et al., 2017), while proximal arm muscle function usually recovers to about 70%‐80% (Gassert & Dietz, 2018). This greater recovery of motor function in proximal limb muscles might be attributable to the activity of the intact or less severely affected RS system.

In a recent imaging study (Schading‐Sassenhausen et al., 2025), the influence of spared spinal tract tissue on mobility and spastic muscle tone was assessed in subjects with SCI. According to this study, substantial damage to the CS tract is associated with a high level of spastic muscle tone. In combination with spared RS projections, a rather moderate spastic muscle tone dominates. Furthermore, the extent of preserved RS tract is associated with a more favourable outcome of mobility following SCI; that is, rudimentary stepping movements become re‐established. This combination is of particular interest, as paresis can be partially compensated for in the presence of spastic muscle tone, thereby enabling some degree of mobility. Thus, the contribution of the RS system on the development of spastic muscle tone allows some mobility to be re‐established at a ‘lower level’ of motor control, that is, by supporting the body during stepping (Dietz & Sinkjaer, 2007). However, in severely affected, immobile individuals, spastic muscle tone can overshoot, requiring therapeutic intervention.

In fact, several studies indicate that an interplay between spared CS and RS fibres can influence the recovery of motor function following stroke or SCI (Sangari & Perez, 2020). In line with this observation, a strengthened RS drive has been suggested to contribute to functional recovery after stroke (Choudhury et al., 2020; Taga et al., 2024). Beneficial effects of RS plasticity were also described for the recovery of hand (Baker & Perez, 2017) and elbow function (Eilfort et al., 2024; Sangari & Perez, 2020) in patients with motor‐incomplete SCI.

**Figure 3:**
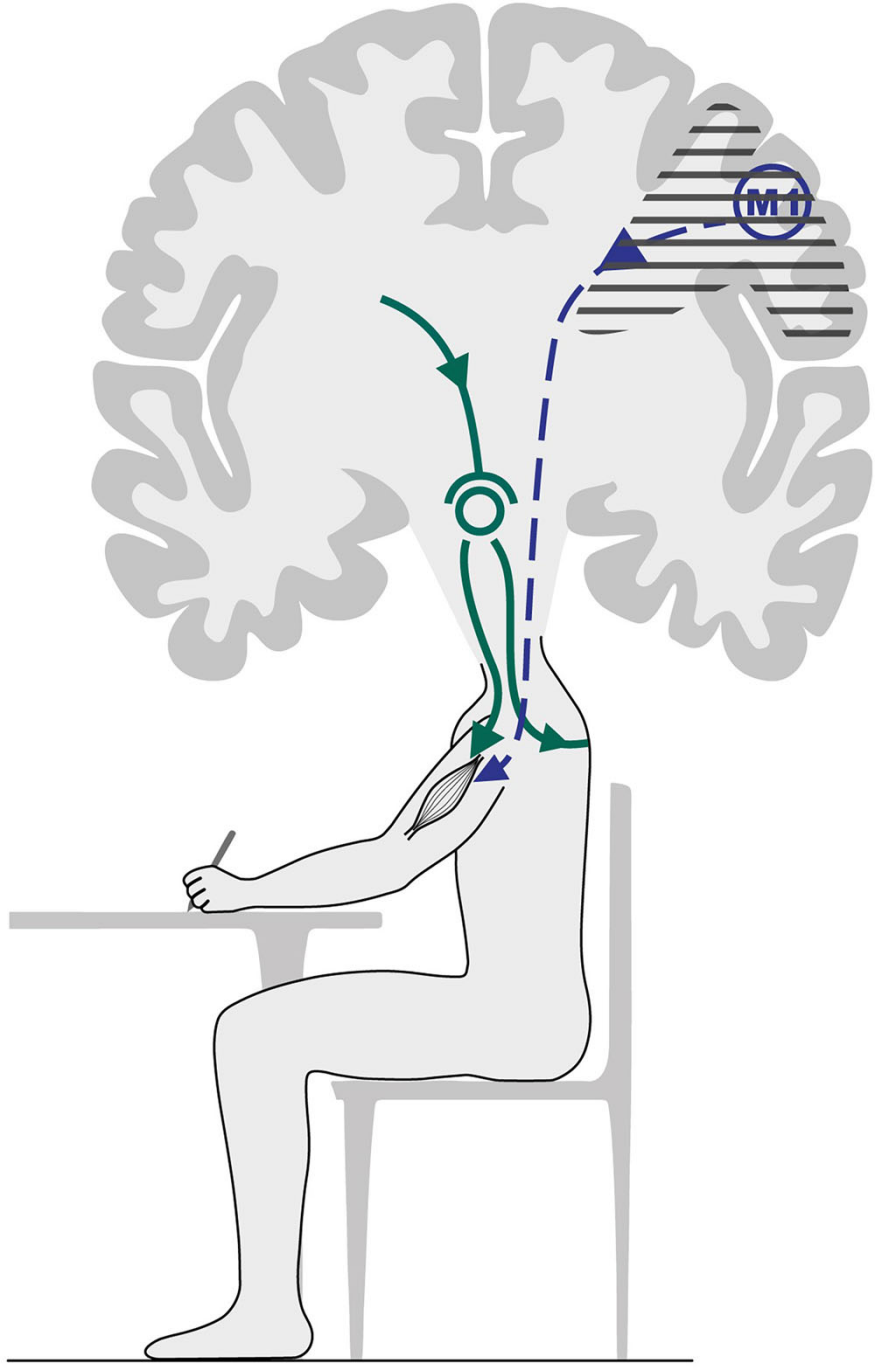
The RS system enhances recovery of function Following a stroke, hand and finger movements on the side contralateral to the brain lesion are impaired. Partial recovery of motor function is suggested to be facilitated by an enhanced drive from brainstem motor centres, particularly the RS system, which partially compensates for the impaired corticospinal drive.

The phylogenetically older motor brainstem centres obviously possess the capacity to play an essential role in promoting recovery of motor limb function. Consequently, the RS system might contribute to the preservation of a basic mobility following stroke or SCI. Indirect evidence suggests that upregulated RS activity can partially compensate for the loss of CS drive, not only in individuals with SCI but also in patients with chronic stroke (Mooney et al., 2025; Fig. 3). However, the movement patterns restored in these patients differ from those observed in healthy individuals (e.g. increased muscle tone, altered muscle synergies). This might be due to the fact that CS and RS pathways differ in their anatomical and physiological properties.

After CNS damage, the neural coupling mechanism is often preserved, although limb coordination is frequently impaired. Following stroke, neural limb coupling has been shown to be partially preserved (Schrasfl‐Altermatt & Dietz, 2016). In most post‐stroke subjects, neural coupling is preserved from the unaffected to the affected limb during bilateral hand movements (Schrafl‐Altermatt & Dietz, 2016). Electrical nerve stimulation applied to the unaffected arm elicits ‘normal’ EMG responses in forearm muscles on both sides, including those of the paretic arm and hand. However, when the nerve of the affected arm is stimulated, only small ipsilateral responses appear. It has been speculated that impaired cortico‐reticular connections induce indirect modulatory effects within the brainstem, thereby disrupting sensorimotor integration of afferent input on the affected side. However, additional mechanisms may contribute to the asymmetric presentation of the coupling response following stroke. The preservation of neural coupling from the unaffected to the affected side has important consequences for motor rehabilitation: bilateral cooperative upper limb training might facilitate the recovery of hand function on the affected side through the influence of the unaffected side via neural limb coupling (Schrafl‐Altermatt & Dietz, 2016).

In conclusion, there is growing evidence that brainstem motor centres, in particular the RS system, play an essential role in both normal and impaired motor control in humans. Recent studies indicate a substantial contribution of the RS system to the recovery of motor function following stroke and SCI. Specifically, the RS system might contribute to improved movement coordination and motor performance in individuals with a CNS injury.

## Additional information

### Competing interests

The authors declare no conflicts of interest.

### Author contributions

Both authors have read and approved the final version of this manuscript and agree to be accountable for all aspects of the work. All persons designated as authors qualify for authorship.

### Funding

The study was supported by the Balgrist Foundation (2021‐0798) and the Swiss National Science Foundation (32003B_208 110).

### Generative AI statement

The authors confirm that no artificial intelligence tools, including large language models (LLMs), were used in the drafting or revision of the manuscript. All content was conceived, written and approved solely by the authors.

## Supporting information


Peer Review History
