# Supplementary material for: Impact of the brainstem in normal and impaired human movement control
Source: J Physiol. 2026 Sep 9;604(18):7559–69. doi: 10.1113/JP290565 (PMC13577698; doi:10.1113/JP290565)
Supplement: Supplementary file 1 [file TJP-604-7559-s001.pdf]

The Journal of Physiology

Impact of the brainstem in normal and impaired human movement control

Volker Dietz and Linard Filli
DOI: 10.1113/JP290565

Corresponding author(s): Volker Dietz (Volker.Dietz@balgrist.ch)

The referees have opted to remain anonymous.

Review Timeline:

Submission Date:	13-Feb-2026
Editorial Decision:	13-Apr-2026
Revision Received:	13-May-2026
Editorial Decision:	01-Jun-2026
Revision Received:	16-Jun-2026
Editorial Decision:	17-Jul-2026
Revision Received:	22-Jul-2026
Accepted:	06-Aug-2026

Senior Editor: Laura Bennet

Reviewing Editor: Ricci Hannah

Transaction Report:

(Note: With the exception of the correction of typographical or spelling errors that could be a source of ambiguity, letters and reports are not edited. Depending on transfer agreements, referee reports obtained elsewhere may or may not be included in this compilation. Referee reports are anonymous unless the Referee chooses to sign their reports.)

Re: JP-TR-2026-290565 "**Involvement of the brainstem in normal and impaired human movement control**" by Volker Dietz and Linard Filli

Dear Dr Dietz,

Thank you for submitting your manuscript to The Journal of Physiology. It has been assessed by a Reviewing Editor and by 2 expert referee(s) and we are pleased to tell you that it is potentially acceptable for publication following satisfactory major revision.

The referee reports are copied at the end of this email.
Please address all the points raised and incorporate all requested revisions or explain in your Response to Referees why a change has not been made. We hope you will find the comments helpful and that you will be able to return your revised manuscript within 2 months. If your article is NOT for a Special Issue, you may have 9 months to revise. If you require an extension, please contact journal staff: jp@physoc.org. Please note that this letter does not constitute a guarantee for acceptance of your revised manuscript.

ABSTRACT FIGURES: Authors are expected to use The Journal's premium BioRender account to create/redraw their Abstract Figures. Information on how to access this account is here:
https://physoc.onlinelibrary.wiley.com/journal/14697793/biorender-access.
This will enable Authors to create and download high-resolution figures. If authors have used the free BioRender service, they can use the instructions provided in the link above to download a high-resolution version suitable for publication.
The link provided should only be used for the purposes of this submission. Authors will be charged for figures created on this account if they are not related to this manuscript submission.

Your revised manuscript should be submitted online using the link in your Author Tasks: Link Not Available. This link is accessible via your account as Corresponding Author; it is not available to your co-authors. If this presents a problem, please contact journal staff (jp@physoc.org). Image files from the previous version are retained on the system. Please ensure you replace or remove any files that are being revised.
If you do not wish to submit a revised version of your manuscript, you must inform our journal staff (jp@physoc.org) or reply to this email to request withdrawal. Please note that a manuscript must be formally withdrawn from the peer review process at one journal before it may be submitted to another journal.

REVISION CHECKLIST:
Check that your Methods section conforms to journal policy: https://jp.msubmit.net/cgi-bin/main.plex?form_type=display_requirements#methods.

Check that data presented conforms to the statistics policy: https://jp.msubmit.net/cgi-bin/main.plex?form_type=display_requirements#statistics.

Upload a full Response to Referees file. To create your 'Response to Referees': copy all the reports, including any comments from the Senior and Reviewing Editors, into a Microsoft Word, or similar, file and respond to each point, using font or background colour to distinguish comments and responses and upload as the required file type.

Please upload two versions of your manuscript text: one with all relevant changes highlighted and one clean version with no changes tracked. The manuscript file should include all tables and figure legends, but each figure/graph should be uploaded as separate, high-resolution files.

You may also upload:
- 'Potential Cover Art' for consideration as the issue's cover image
- Appropriate Supporting Information (Video, audio or data set: see https://jp.msubmit.net/cgi-bin/main.plex?form_type=display_requirements#supp).

IMPORTANT POINTS TO NOTE WHEN REVISING YOUR MANUSCRIPT:
LANGUAGE EDITING AND SUPPORT FOR PUBLICATION: If you would like help with English language editing, or other article preparation support, Wiley Editing Services offers expert help, including English Language Editing, as well as translation, manuscript formatting, and figure formatting at www.wileyauthors.com/eeo/preparation. You can also find resources for Preparing Your Article for general guidance about writing and preparing your manuscript at www.wileyauthors.com/eeo/prepresources.

TRANSPARENT PEER REVIEW POLICY: To improve the transparency of its peer review process, The Journal of Physiology publishes online as supporting information the peer review history of all articles accepted for publication. Readers will have access to decision letters, including Editors' comments and referee reports, for each version of the manuscript, as well as any author responses to peer review comments. Referees can decide whether or not they wish to be named on the peer review history document.

We look forward to receiving your revised submission.

If you have any queries, please reply to this email and we will be pleased to advise.

Yours sincerely,

Laura Bennet
Senior Editor
The Journal of Physiology

REQUIRED ITEMS

- Author profile(s) must be uploaded via the submission form. Authors should submit a short biography (no more than 100 words for one author or 150 words in total for two authors) and a portrait photograph of the two leading authors on the paper. These should be uploaded and clearly labelled together in a Word document with the revised version of the manuscript. Any standard image format for the photograph is acceptable, but the resolution should be at least 300 DPI and preferably more. A group photograph of all authors is also acceptable, providing the biography for the whole group does not exceed 150 words.

EDITOR COMMENTS

Reviewing Editor:

Both reviewers find the topic important and the review promising, but they raise several concerns regarding accuracy, clarity, and interpretation. In particular, there are instances of mis-citation and imprecise description of prior work, as well as overinterpretation of evidence, especially in attributing findings to reticulospinal mechanisms versus alternative explanations (e.g., spinal circuitry). The reviewers also encourage clearer justification of key claims and better integration of relevant recent literature.

REFEREE COMMENTS

Referee #1:

This is an interesting brief review of evidence for the involvement of brainstem pathways in human motor control. I have several detailed comments where the article could be improved.

Line 72, Do we actually know much about the contribution of CS and RS systems to different tasks? The paper cited presented work in anaesthetised animals, so is not really an appropriate support for this statement.

Line 93, the available evidence from the paper by Riddle et al (2009) cited shows that the RS system projects to both distal and proximal muscles. Indeed, in that paper connections seemed to be stronger to intrinsic hand muscles than more proximal muscle groups.

Line 108, the study cited by Glover & Baker (2020) was performed in monkeys, not in humans as stated. A later paper by the same authors (Glover & Baker, 2022) looked at force control (also in monkey), and might be the reference that the authors were thinking of.

Line 110, Baker & Perez (2017) looked at power grasp, not at tasks involving axial and proximal muscles.

Line 124, 'develops or the cost of extrapyramidal pathways' is hard to understand, is 'develops at the expense of extrapyramidal pathways' what was meant? Is there any evidence for this statement? The paper cited by Nakajima et al

looked at monosynaptic vs disynaptic corticospinal inputs to motoneurons, with the disynaptic connections going via propriospinal interneurons. That latter pathway cannot reasonably be called 'extrapyramidal': it still involves the corticospinal tract. Does any evidence actually show a decrease in extrapyramidal pathways like the RS system concomitant with the known increase in corticospinal pathways in the phylogeny of primates up to humans?

Line 172, it is not reasonable to conclude that because a response 'depends on the level of background muscle activation', it 'depends on the force and movement velocity exerted by the hands'. A dependence of response on background activation is expected from any input to motoneurons, a phenomenon known as automatic gain scaling (see Matthews, 1986, J Physiol 374: 73-90, although there are earlier papers).

Line 195, although skilled hand movements require cortico-motoneuronal connections, there is also evidence for brainstem contributions. As mentioned above, the RS system makes mono- and di-synaptic connections to motoneurons innervating intrinsic hand muscles (Riddle et al, 2009). Cells in the reticular formation modulate their discharge during precise finger movements just as much, if not more, than cells in the hand representation of M1 (Soteropoulos et al, 2012, J Physiol 590, 4011).

Line 198, also 275-278, another candidate system that could be considered for cross-limb coupling is commissural interneurons, which are known to exist in primate cervical spinal cord (Soteropoulos et al, 2013, J Neurosci 33,9614).

Line 280, some explanation is required as to how the responses are similar to loud tones and to contralateral nerve stimulation.

Line 360-361: The explanation, 'due to an impaired processing of afferent input from the affected side', really just restates the observation. Could the authors speculate on the underlying neural mechanisms, and could this fit with a role for the RS system?

Referee #2:

I have the following comments:

1) I could not locate the scatter plot data for Figure 1 in the cited reference (Thomas et al., 2018), and key details of this figure are not described. For example, in the two scatter plots, does the background EMG (x-axis) correspond to activity on the stimulated side? (while the reflex magnitude reflects responses on both the stimulated and non-stimulated sides). Given that the task involves interlimb coordination, does this also imply that EMG activity on the non-stimulated side covaries with that on the stimulated side as a function of task demands?

In either case, the observed relationship in Figure 1 may simply indicate that reflex amplitude on each side scales with its respective level of background EMG. This would be expected, as increased motoneuron excitability (reflected in higher background EMG) typically leads to larger reflex responses. From this perspective, the result appears consistent with modulation at the level of local spinal circuitry and motoneuron excitability, rather than providing clear evidence for a brainstem-mediated mechanism. Please elaborate more on this.

2) One of main comments is regarding the attribution of inter-limb coordination functions to reticulospinal tract. For example:

Accordingly, upper limb swing during stepping appears to be part of locomotor activity, reflecting the persistence of a quadrupedal organization of bipedal gait (Dietz, 2002), This is suggested to be under the control of the brainstem, presumably the RS system.

Or: Muscle responses to ES of unaffected limb of stroke subjects: 'Normal' responses appear in muscles of the contralateral,

paretic limb.

However, it is not clear whether these findings distinguish between contributions from local spinal circuitry (e.g., intersegmental or commissural interneurons) and brainstem-centered mechanisms in mediating inter-limb coordination. Please elaborate on this distinction and clarify how the reported experiments specifically support a role for the reticular formation in these processes. In addition, please indicate the extent to which this interpretation is directly supported by the data versus remaining speculative.

3) Please discuss recent findings in rodents and humans regarding the role of medullary reticular formation in forelimb/hand motor control such as Falasconi et al, Nature 2025; Yang et al, Cell 2023; Jindal et al, PNAS, 2026; Esposito et al, Nature, 2014. / There is growing neuroanatomical and neurophysiological evidence that the RS system has a key function in motor control of vertebrates.... /

Please also discuss recent studies in humans examining the role of medulla in hand force control in humans such as Jindal et al, PNAS, 2026. / Also Recent studies in humans indicate an involvement of the RS in force control (Glover & Baker, 2020)... /

4) It can be concluded that the presence of sufficient spastic muscle tone allows a mobility on a 'lower level' of motor control.

While spasticity in the lower limb can, in some cases, contribute functionally to weight-bearing and aspects of gait, its presence in the upper limb is generally counterproductive. Accordingly, many rehab programs prioritize reducing upper-limb spasticity. Once spastic hypertonia is adequately managed, patients are better able to engage in and benefit from standard rehabilitative interventions. Please moderate and elaborate on beneficial effects for upper vs lower limb.

5) It is argued that the similarity between EMG responses evoked by ulnar nerve stimulation and those elicited by loud acoustic stimuli (Dietz et al., 2024) provides key support for a role of RST in interlimb coordination. Please elaborate on what specific features of the EMG responses underpin this comparison, beyond the presence of a bilateral activation pattern. Thank you!

END OF COMMENTS

Dear Editors,

many thanks for the feedback with the notion that our manuscript is potentially acceptable for publication. We are grateful for the comments of the Editor and the Reviewers: All comments were constructive and helpful to improve quality of the manuscript.

EDITOR COMMENTS

Reviewing Editor:

Both reviewers find the topic important and the review promising, but they raise several concerns regarding accuracy, clarity, and interpretation. In particular, there are instances of mis-citation and imprecise description of prior work, as well as overinterpretation of evidence, especially in attributing findings to reticulospinal mechanisms versus alternative explanations (e.g., spinal circuitry). The reviewers also encourage clearer justification of key claims and better integration of relevant recent literature.

----------------- We acknowledge all comments and have addressed shortcomings in the manuscript. We have tried to revise the manuscript accordingly.

REFEREE COMMENTS

Referee #1:

This is an interesting brief review of evidence for the involvement of brainstem pathways in human motor control. I have several detailed comments where the article could be improved.

Line 72, Do we actually know much about the contribution of CS and RS systems to different tasks? The paper cited presented work in anaesthetised animals, so is not really an appropriate support for this statement.

We acknowledge this comment and correct it appropriately (without reference): '…little is known about the relative contributions of CS and RS to movement control. It is assumed to vary and most likely depends on the motor task to be performed.'

Line 93, the available evidence from the paper by Riddle et al (2009) cited shows that the RS system projects to both distal and proximal muscles. Indeed, in that paper connections seemed to be stronger to intrinsic hand muscles than more proximal muscle groups.

We performed corresponding corrections '…as well as upper limb function (Riddle et al. 2009) by the RS was described.

Line 108, the study cited by Glover & Baker (2020) was performed in monkeys, not in humans as

stated. A later paper by the same authors (Glover & Baker, 2022) looked at force control (also in monkey), and might be the reference that the authors were thinking of.

Thanks! We have changed the reference appropriately to Glover & Baker, 2022.

Line 110, Baker & Perez (2017) looked at power grasp, not at tasks involving axial and proximal muscles.

We have made appropriate corrections and now state "Recent studies in humans indicate an involvement of the RS in force control (Glover & Baker, 2022) and facilitation of explosive movements (Eilfort & Filli, 2025). RS drive appears to be particularly strong to upper limb flexors, including those mediating power grasp tasks (Baker and Perez, 2017), and lower limb extensors (Eilfort et al., 2025)

Line 124, 'develops or the cost of extrapyramidal pathways' is hard to understand, is 'develops at the expense of extrapyramidal pathways' what was meant? Is there any evidence for this statement? The paper cited by Nakajima et al looked at monosynaptic vs disynaptic corticospinal inputs to motoneurons, with the disynaptic connections going via propriospinal interneurons. That latter pathway cannot reasonably be called 'extrapyramidal': it still involves the corticospinal tract. Does any evidence actually show a decrease in extrapyramidal pathways like the RS system concomitant with the known increase in corticospinal pathways in the phylogeny of primates up to humans?

This addresses an interesting point where we are not completely in line with the Reviewer: Our statement refers to fig.12 C & D of the paper of Nakajima et al 2000. According to this figure, the phylogenetic development of the CST parallels the dexterity of the upper limb. In contrast, indirect projections via propriospinal neurons, including those of brainstem nuclei (LRN tract), seem to regress with increasing dexterity. Therefore, we would like to leave the statement and to include the sentence (proposed by the reviewer) '..develops at the expense of extrapyramidal pathways'. Is this ok? Otherwise we are prepared to omit this sentence.

Line 172, it is not reasonable to conclude that because a response 'depends on the level of background muscle activation', it 'depends on the force and movement velocity exerted by the hands'. A dependence of response on background activation is expected from any input to motoneurons, a phenomenon known as automatic gain scaling (see Matthews, 1986, J Physiol 374: 73-90, although there are earlier papers).

We agree with this statement and have changed the sentence appropriately. "…which amplitudes depend on the level of background muscle activation (automatic reflex gain scaling; cf Matthews 1986). In such a condition, the level background muscle activity on both sides depends on the force and movement velocity exerted by the hands (Thomas et al. 2018)."

Line 195, although skilled hand movements require cortico-motoneuronal connections, there is also evidence for brainstem contributions. As mentioned above, the RS system makes mono- and di-synaptic connections to motoneurons innervating intrinsic hand muscles (Riddle et al, 2009). Cells in the reticular formation modulate their discharge during precise finger movements just as much, if not more, than cells in the hand representation of M1 (Soteropoulos et al, 2012, J Physiol 590, 4011).

We acknowledge this comment, although clinical evidence indicates a corticospinal dominance. As outlined in the third part of the review, recovery of motor function after a stroke is typically more pronounced for proximal upper limb muscles (up to 70 to 80 %) compared to hand/finger muscles (about 20% recovery).--i.e., there are pronounced differences.However, we have incorporated the reviewer's statement as follows "…..mainly under the control of the corticomotoneuronal system, although RS projections are known to contribute to fine finger movements (Soteropoulos et al. 2012) .

Line 198, also 275-278, another candidate system that could be considered for cross-limb coupling is commissural interneurons, which are known to exist in primate cervical spinal cord (Soteropoulos et al, 2013, J Neurosci 33,9614).

This is indeed an interesting point. We have included this aspect in Table 1 and in the respective paragraph: "Based on findings in non-human primates, commissural interneurons in the cervical cord might contribute to the neural interlimb coupling (Soteropoulos et al. 2013)."

Line 280, some explanation is required as to how the responses are similar to loud tones and to contralateral nerve stimulation.

We clarified this issue in the revised version as follows: "The muscle responses to loud tones, known to activate the RS system, were identical in their size, distribution and configuration to the contralateral reflex responses elicited by ipsilateral nerve stimulation."

Line 360-361: The explanation, 'due to an impaired processing of afferent input from the affected side', really just restates the observation. Could the authors speculate on the underlying neural mechanisms, and could this fit with a role for the RS system?

This represents a quite interesting point. As proposed by the Reviewer we speculate on the neural mechanism underlying this observation:
"It is speculated that the afferent volley elicited by the electrical nerve stimulus might not be integrated in the RS system due to impaired cortico-reticulospinal connections."

Referee #2:

I have the following comments:

1) I could not locate the scatter plot data for Figure 1 in the cited reference (Thomas et al., 2018), and key details of this figure are not described. For example, in the two scatter plots, does the background EMG (x-axis) correspond to activity on the stimulated side? (while the reflex magnitude reflects responses on both the stimulated and non-stimulated sides). Given that the task involves interlimb coordination, does this also imply that EMG activity on the non-stimulated side covaries with that on the stimulated side as a function of task demands?

In either case, the observed relationship in Figure 1 may simply indicate that reflex amplitude on each side scales with its respective level of background EMG. This would be expected, as

increased motoneuron excitability (reflected in higher background EMG) typically leads to larger reflex responses. From this perspective, the result appears consistent with modulation at the level of local spinal circuitry and motoneuron excitability, rather than providing clear evidence for a brainstem-mediated mechanism. Please elaborate more on this.

The scatter plot shown in Fig. 1 is derived from figure 3 of Thomas et al., 2018. The volunteers performed an 'opening a bottle' task with both hands on a device that enabled movements with changing forces and velocities. In such a task, the level of background EMG has to be the same on both sides. The amplitude of the reflex response again corresponds to the level of background EMG on both sides, known as automatic reflex gain scaling (described for the stimulated side by Matthews 1986). The essential aspect of this figure is that the same EMG pattern appears on both sides, i.e., also on the non-stimulated side, reflecting the neural coupling mechanism during cooperative hand movements. This figure does not explain the neural mechanism mediating the neural coupling, which was not yet known at the time of this publication.

In the revised version of the manuscript we explain this figure with the scatter plots in more detail: "'… a unilateral distal arm nerve stimulation is followed by EMG responses in the forearm muscles of both sides which amplitudes depend on the level of background muscle activity (automatic reflex gain scaling, (Matthews, 1986)). In such a condition, the background EMG (same level on both sides) reflects the automatic matching of the force and movement velocity exerted by the opening hand with the force and velocity exerted by the opposite, holding hand. This occurs with the goal: two hands, one action (Thomas *et al.*, 2018)'

2) One of main comments is regarding the attribution of inter-limb coordination functions to reticulospinal tract. For example:

Accordingly, upper limb swing during stepping appears to be part of locomotor activity, reflecting the persistence of a quadrupedal organization of bipedal gait (Dietz, 2002), This is suggested to be under the control of the brainstem, presumably the RS system.

Or: Muscle responses to ES of unaffected limb of stroke subjects: 'Normal' responses appear in muscles of the contralateral, paretic limb.

However, it is not clear whether these findings distinguish between contributions from local spinal circuitry (e.g., intersegmental or commissural interneurons) and brainstem-centered mechanisms in mediating inter-limb coordination. Please elaborate on this distinction and clarify how the reported experiments specifically support a role for the reticular formation in these processes. In addition, please indicate the extent to which this interpretation is directly supported by the data versus remaining speculative.

In fact, this is an important point. Our data suggest that the interlimb coordination reflected in the neural coupling is likely mediated by the brainstem, most likely by the reticular formation. Our arguments for this assumption are listed in Table 1. However, we cannot exclude that reticulospinal drive is distributed, via spinal interneurons, to both sides of the spinal cord, and that spinal interneurons might contribute to the neural coupling. Reviewer 1 had a similar

comment (lines 275-278). We have included this aspect in the revised version of the manuscript and in Table 1.
However, the main neural factors mediating neural coupling (cf Table 1) are described in the manuscript (e.g. similarity of the EMG responses to acoustic and electrical nerve stimuli (Dietz et al. 2024)).

3) Please discuss recent findings in rodents and humans regarding the role of medullary reticular formation in forelimb/hand motor control such as Falasconi et al, Nature 2025; Yang et al, Cell 2023; Jindal et al, PNAS, 2026; Esposito et al, Nature, 2014. / There is growing neuroanatomical and neurophysiological evidence that the RS system has a key function in motor control of vertebrates.... /

Please also discuss recent studies in humans examining the role of medulla in hand force control in humans such as Jindal et al, PNAS, 2026. / Also Recent studies in humans indicate an involvement of the RS in force control (Glover & Baker, 2020)... /

We fully agree with the Referee that animal and human studies support the assumption that the RS system has a key function in motor control (see Abstract, last sentence and Conclusions of part 1 and 2). We have added the findings of the respective references in the revised manuscript. The additional references provided by the referee are included in the basic part 1 (p3, last para): The references Jindal et al. 2026; Esposito et al. 214; p4; 2nd para, and Glover and Baker 2022, p4, last para.
This reads now as follows in the text (line 97): The medullary part of the reticular formation seems to play a key role in motor control of the upper limb in animals (Esposito et al., 2014; Zhang et al., 2024; Falasconi et al., 2025) and humans (Jindal et al., 2026)."

4) It can be concluded that the presence of sufficient spastic muscle tone allows a mobility on a 'lower level' of motor control.

While spasticity in the lower limb can, in some cases, contribute functionally to weight-bearing and aspects of gait, its presence in the upper limb is generally counterproductive. Accordingly, many rehab programs prioritize reducing upper-limb spasticity. Once spastic hypertonia is adequately managed, patients are better able to engage in and benefit from standard rehabilitative interventions. Please moderate and elaborate on beneficial effects for upper vs lower limb.

Basically, we agree with this comment. After a stroke most patients can only perform stepping movements due to the support of body weight by a spastic muscle tone. Although spastic muscle tone varies considerably between patients, we cannot fully agree with the notion that upper limb spasticity is mostly 'counterproductive'. With the flexed position of the spastic arm, patients can usually use the arm to carry on objects. In our hospital we treat spastic muscle tone –if required—by physiotherapeutic measures. Only in rare cases, spastic muscle tone is treated by Botox injections. Therefore, we cannot see fundamental differences between upper and lower limb spasticity.

5) It is argued that the similarity between EMG responses evoked by ulnar nerve stimulation and those elicited by loud acoustic stimuli (Dietz et al., 2024) provides key support for a role of RST in interlimb coordination. Please elaborate on what specific features of the EMG responses underpin this comparison, beyond the presence of a bilateral activation pattern.

Thank you!

We are grateful for this useful comment. The fact that the EMG responses to loud acoustic stimuli (known to activate the RS system) were identical (in amplitude, configuration, and distribution, cf Dietz et al, 2024) with those elicited by unilateral nerve stimulation, provides indirect evidence that these responses are generated in the same neural structure i.e. the RS.
We have now extended this section (main arguments summarized in Table 1) in the revised manuscript.

Dear Professor Dietz,

Re: JP-TR-2026-290565R1 "**Impact of the brainstem in normal and impaired human movement control**" by Volker Dietz and Linard Filli

Thank you for submitting your manuscript to The Journal of Physiology. It has been assessed by a Reviewing Editor and by 2 expert referees and we are pleased to tell you that it is acceptable for publication following satisfactory revision.

The referee reports are copied at the end of this email.

Please address all the points raised and incorporate all requested revisions or explain in your Response to Referees why a change has not been made. We hope you will find the comments helpful and that you will be able to return your revised manuscript within 4 weeks. If you require longer than this, please contact journal staff: jp@physoc.org.

Your revised manuscript should be submitted online using the link in your Author Tasks: Link Not Available. This link is accessible via your account as Corresponding Author; it is not available to your co-authors. If this presents a problem, please contact journal staff (jp@physoc.org). Image files from the previous version are retained on the system. Please ensure you replace or remove any files that are being revised.

If you do not wish to submit a revised version of your manuscript, you must inform our journal staff (jp@physoc.org) or reply to this email to request withdrawal. Please note that a manuscript must be formally withdrawn from the peer review process at one journal before it may be submitted to another journal.

TRANSPARENT PEER REVIEW POLICY: To improve the transparency of its peer review process, The Journal of Physiology publishes online as supporting information the peer review history of all articles accepted for publication. Readers will have access to decision letters, including Editors' comments and referee reports, for each version of the manuscript, as well as any author responses to peer review comments. Referees can decide whether or not they wish to be named on the peer review history document.

ABSTRACT FIGURES: Authors may use The Journal's premium BioRender account to create/redraw their Abstract Figures (and any other suitable schematic figure). Information on how to access this account is here: https://physoc.onlinelibrary.wiley.com/journal/14697793/biorender-access.

This will enable Authors to create and download high-resolution figures. If authors have used the free BioRender service, they can use the instructions provided in the link above to download a high-resolution version suitable for publication. The link provided should only be used for the purposes of this submission. Authors will be charged for figures created on this account if they are not related to this manuscript submission.

LANGUAGE EDITING AND SUPPORT FOR PUBLICATION: If you would like help with English language editing, or other article preparation support, Wiley Editing Services offers expert help, including English Language Editing, as well as translation, manuscript formatting, and figure formatting at www.wileyauthors.com/eeo/preparation. You can also find resources for Preparing Your Article for general guidance about writing and preparing your manuscript at www.wileyauthors.com/eeo/prepresources.

REVISION CHECKLIST: Upload a full Response to Referees file. To create your 'Response to Referees' copy all the reports, including any comments from the Senior and Reviewing Editors, into a Microsoft Word, or similar, file and respond to each point, using font or background colour to distinguish comments and responses and upload as the required file type.

Please upload two versions of your manuscript text: one with all relevant changes highlighted and one clean version with no changes tracked. The manuscript file should include all tables and figure legends, but each figure/graph should be uploaded as separate, high-resolution files.

You may also upload:

- 'Potential Cover Art' for consideration as the issue's cover image
- Appropriate Supporting Information (Video, audio or data set: see https://jp.msubmit.net/cgi-bin/main.plex?form_type=display_requirements#supp).

We look forward to receiving your revised submission.

If you have any queries, please reply to this email and we will be pleased to advise.

Yours sincerely,

Laura Bennet
Senior Editor

REQUIRED ITEMS

- Authors are required to fully disclose the use of any Generative AI (GAI) tools used in the preparation of their manuscript. This disclosure should also be included in the 'Methods' section (or a suitable location for article types not requiring Methods), and include the name(s), model and version of AI technology used, and exact details on the purpose for, and methods of, its use.

If no AI tool has been used, this should also be specifically stated within the manuscript.

EDITOR COMMENTS

Reviewing Editor:

Thank you for your revised manuscript. The referees agree that the paper has improved, but a few issues remain. Please address the remaining mechanistic and citation concerns raised by Referee 1, particularly around the interpretation of cortico-reticulospinal/reticulospinal pathways and the evidence for brainstem involvement. Please also moderate the claims about beneficial effects of upper limb spasticity, as requested by Referee 2. Finally, please carry out a careful language edit throughout.

REFEREE COMMENTS

Referee #1:

The manuscript has been improved since the first version, with responses to most of the two referees' comments incorporated into the paper. There remain a few issues.

Line 114: although this sentence has been altered, it still miscites the Glover & Baker paper, which was not carried out in humans ('Recent studies in humans...').

Line 130: '...develops at the expense of extrapyramidal pathways'. The response shows a misunderstanding of the connectivity of propriospinal interneurons as illustrated in the Nakajima paper. The LRN does not project to propriospinal neurons, but receives collateral input from them. All of the Nakajima paper, including Fig. 12CD, involves corticospinal projections, either direct to motoneurons or indirectly via propriospinal interneurons. Citation of this paper cannot therefore be used to support the statement on extrapyramidal pathways.

Line 290: the authors have clarified that responses to loud sounds were identical in 'size, distribution and configuration' to those following contralateral nerve stimulation. I do not find this convincing evidence:

Size. Two responses could be carried by the same pathway, and be of different size (because one is caused by more powerful activation of that pathway than the other). Equally, responses of equal amplitude could be generated by different pathways.

Distribution. Two responses could engage different cells within the same pathway and produce different patterns of activity across muscles. Equally, responses carried over different pathways could generate similar patterns. In particular, it should be possible to mimic any pattern of activity across muscles using the highly fractionated corticospinal tract.

Configuration. I am not at all clear what this term refers to.

Is there any actual evidence for similarity of pathway? Either way, this section should be rewritten, along with line 237 which deals with the same issue.

Line 245: This sentence gives the impression that the CRS is a separate system in contrast to the RS ('besides the RS system...'). But the RS is controlled by the cortex; we can refer to this in shorthand as the cortico-reticulospinal system (CRS). Line 271 also seems to have the same problem, of viewing the CRS as a distinct system.

Line 363-4: There is no verb in this sentence.

Line 371. It is speculated that an afferent volley might fail to be effective, because of reduced cortico-reticulospinal drive. However, there are direct projections from the cuneate nucleus to the reticular formation; there is no need for afferent input to pass via the cortex, and hence no need for a dependence on cortico-reticular connections.

Line 376: 'growing for' -> 'growing evidence for'.

As a general comment the writing could be improved throughout: there are multiple instances of slightly strange or awkward English usage. The authors should ask a native English speaker to help improve the language - or perhaps pass it through an AI tool. This would improve clarity for the reader.

Referee #2:

The authors have now adequately addressed most of my comments from the previous revision. I only have one remaining comment regarding a previously raised point that was not fully addressed, as outlined below.

I agree with the authors that currently there is no effective treatment for spasticity, and BoNT-A injection in many cases are not are not preferable to physiotherapy. However, the lack of an effective treatment does not make the upper limb spasticity a beneficial outcome. Having high flexor contractions limits mobility of various joints and impair functional abilities. Many studies (please find a few example references below) have reported that upper limb spasticity contributes to impairment of movement function as well as limitation of activity, and that therapy intensity can improve outcomes after spasticity treatment.

As this review focuses on the role of the RST in motor control rather than presenting a perspective article on spasticity, I would encourage the authors to moderate their claims regarding the beneficial effects of spasticity on movement, as such conclusions are not currently well supported by the substantial body of evidence in the literature:

- Welmer et al., 2006, Cerebrovascular Diseases (Spasticity and its association with functioning and health-related quality of life 18 months after stroke)

- Kong et al., 2010, Journal of Rehabilitation Medicine (Symptomatic upper limb spasticity in patients with chronic stroke attending a rehabilitation clinic: frequency, clinical correlates and predictors)

- Pundik et al., 2019, Annals of Physical and Rehabilitation Medicine (Association of spasticity and motor dysfunction in chronic stroke)

- Bhakta et al., 2000, J Neurol Neurosurg Psychiatry

- Brashear et al., 2002, New England Journal of Medicine

- Sun et al., 2010, Neurorehabilitation and Neural Repair

- Devier et al., 2017, Toxins

- Franck et al., 2021, NeuroRehabilitation

- Demetrios et al., 2014, Journal of Rehabilitation Medicine

END OF COMMENTS

Point by point response

Manuscript ID: JP-TP-2026- 290565R1

Reviewing Editor:
Thank you for your revised manuscript. The referees agree that the paper has improved, but a few issues remain. Please address the remaining mechanistic and citation concerns raised by Referee 1, particularly around the interpretation of cortico-reticulospinal/reticulospinal pathways and the evidence for brainstem involvement. Please also moderate the claims about beneficial effects of upper limb spasticity, as requested by Referee 2. Finally, please carry out a careful language edit throughout.

We thank the editor and referees for their constructive comments. We have addressed the remaining issues in the point-by-point responses and have incorporated the respective feedback into the manuscript. Additionally, we have carefully edited the English language throughout the manuscript using AI-based language editing tools. We hope that the revised manuscript is now suitable for publication in The Journal of Physiology.

Referee 1:

The manuscript has been improved since the first version, with responses to most of the two referees' comments incorporated into the paper. There remain a few issues.

Line 114: although this sentence has been altered, it still miscites the Glover & Baker paper, which was not carried out in humans ('Recent studies in humans...').

We thank the reviewer for pointing out this mis-citation. We have corrected it in the revised manuscript.

Line 130: '...develops at the expense of extrapyramidal pathways'. The response shows a misunderstanding of the connectivity of propriospinal interneurons as illustrated in the Nakajima paper. The LRN does not project to propriospinal neurons, but receives collateral input from them. All of the Nakajima paper, including Fig. 12CD, involves corticospinal projections, either direct to motoneurons or indirectly via propriospinal interneurons. Citation of this paper cannot therefore be used to support the statement on extrapyramidal pathways.

We revised the relevant section of the manuscript and removed the citation Nakajima et al., 2000. The text now reads: "In conclusion, the CS tract has undergone considerable evolutionary development and become the dominant motor system in primates. However, accumulating evidence over recent years indicates that the RS system plays a fundamental role in basic aspects of motor control, not only in vertebrates but also in humans."

Line 290: the authors have clarified that responses to loud sounds were identical in 'size, distribution and configuration' to those following contralateral nerve stimulation. I do not find this convincing evidence:

Size. Two responses could be carried by the same pathway, and be of different size (because one is caused by more powerful activation of that pathway than the other). Equally, responses of equal amplitude could be generated by different pathways.

We agree with this comment. Depending on the stimulus domain and intensity, response size can differ between ES and LAS. More convincing evidence for the similarity of the responses, and the suggested common origin, are the analogous response components and their respective timing (see responses below).

Distribution. Two responses could engage different cells within the same pathway and produce different patterns of activity across muscles. Equally, responses carried over different pathways could generate similar patterns. In particular, it should be possible to mimic any pattern of activity across muscles using the highly fractionated corticospinal tract.

Configuration. I am not at all clear what this term refers to.

Is there any actual evidence for similarity of pathway? Either way, this section should be rewritten, along with line 237 which deals with the same issue.

We revised the relevant sections to provide a stronger rationale for the hypothesis that responses to electrical (ES) and loud acoustic (LAS) stimulation are mediated by a common neural pathway. We acknowledge that the evidence supporting this assumption is indirect. Nevertheless, we argue that the convergence of findings across studies provides support for the assumption that ES- and LAS-evoked responses are mediated by the same brainstem structure, most likely the reticulospinal system (for technical details we refer in this 'Topical Review' to a paper published recently: Dietz et al., 2024; https://doi.org/10.1113/JP28540).

The essential aspects for our (published) view:

- LAS are known to activate neurons in the reticular formation, whereas neurons in the motor cortex are suppressed (Furubayashi et al., 2000; Tapia et al., 2022).
- LAS evoke EMG responses (i.e., coupling-like responses) that can be separated from auditory vestibular-evoked responses (VEMPs) and the acoustic startle response (Dietz et al., 2024). The fact that coupling-like responses are evoked by LAS suggest that these responses are mediated by brainstem circuits, rather than spinal or cortical networks (Furubayashi et al., 2000).
- Unilateral ES to the right ulnar nerve evokes bilateral EMG responses (i.e., coupling responses) in muscles of moving limbs. These responses have been reported and analysed in several previous papers (see references). ES-evoked responses exhibit identical response configuration, i.e. duration of responses and negative-to-positive peak characteristics as that of LAS-induced responses.
- Peak latencies do not differ between responses to LAS and ES, consistent with the finding that ES, applied 10 ms prior to LAS, converge within brainstem structures (German et al., 2021).
- Non-parametric statistical mapping revealed no significant difference in the time-series characteristics of EMG responses evoked by LAS and ES in the forearm extensors contralateral to ulnar nerve stimulation. Consistent with this findings, Bayes factor analysis provided anecdotal evidence for the similarity of coupling-like and coupling response latencies for both the positive and negative peaks.
- The provided findings are consistent with the assumption of a common origin of the ES- and LAS-evoked responses within the brainstem (Dietz et al. 2024).

Line 245: This sentence gives the impression that the CRS is a separate system in contrast to the RS ('besides the RS system...'). But the RS is controlled by the cortex; we can refer to this in shorthand as the cortico-reticulospinal system (CRS). Line 271 also seems to have the same problem, of viewing the CRS as a distinct system.

We adapted this statement (also the one in line 271) to clarify the issue. It is now stated that: "Furthermore, findings from experimental CS lesions indicate that certain aspects of corticomotor control are mediated through the cortico-reticulospinal (CRS) system, and that the CRS pathway can serve to compensate for some impaired CS function (Lawrence & Kuypers, 1968; Asboth et al., 2018).
Furthermore, it is mentioned that: "The reticular formation is regulated through extensive cortico-reticular connections providing the brainstem with motor-related information (Darling et al., 2018; Fisher et al., 2021). Therefore, the CRS system is considered to represent an important part of the motor control system across vertebrate species.

Line 363-4: There is no verb in this sentence.

Thank you, we corrected this mistake.

Line 371. It is speculated that an afferent volley might fail to be effective, because of reduced cortico-reticulospinal drive. However, there are direct projections from the cuneate nucleus to the reticular formation; there is no need for afferent input to pass via the cortex, and hence no need for a dependence on cortico-reticular connections.

We agree with the reviewer. Nevertheless, impaired cortico-reticular connections may alter neural excitability within the brainstem, thereby affecting bulbar sensorimotor integration and the manifestation of the coupling response in stroke patients. We have therefore reformulated this hypothesis more cautiously in the revised manuscript. The text now reads: “It is speculated that the afferent volley, elicited by electrical nerve stimulation on the affected side, may not be adequately integrated within RS circuits, as impaired cortico-reticulospinal connections may lead to dysregulation of brainstem neuronal networks."

Line 376: 'growing for' -> 'growing evidence for'.
We corrected this mistake.

As a general comment the writing could be improved throughout: there are multiple instances of slightly strange or awkward English usage. The authors should ask a native English speaker to help improve the language - or perhaps pass it through an AI tool. This would improve clarity for the reader.

We have improved the language throughout the manuscript using AI-assisted editing tools (ChatGPT), as suggested. We believe that these revisions have enhanced the clarity and readability of the manuscript.

Referee 2:

The authors have now adequately addressed most of my comments from the previous revision. I only have one remaining comment regarding a previously raised point that was not fully addressed, as outlined below.

I agree with the authors that currently there is no effective treatment for spasticity, and BoNT-A injection in many cases are not are not preferable to physiotherapy. However, the lack of an effective treatment does not make the upper limb spasticity a beneficial outcome. Having high flexor contractions limits mobility of various joints and impair functional abilities. Many studies (please find a few example references below) have reported that upper limb spasticity contributes to impairment of movement function as well as limitation of activity, and that therapy intensity can improve outcomes after spasticity treatment.
As this review focuses on the role of the RST in motor control rather than presenting a perspective article on spasticity, I would encourage the authors to moderate their claims regarding the beneficial effects of spasticity on movement, as such conclusions are not currently well supported by the substantial body of evidence in the literature:

- Welmer et al., 2006, Cerebrovascular Diseases (Spasticity and its association with functioning and health-related quality of life 18 months after stroke)

- Kong et al., 2010, Journal of Rehabilitation Medicine (Symptomatic upper limb spasticity in patients with chronic stroke attending a rehabilitation clinic: frequency, clinical correlates and predictors)

- Pundik et al., 2019, Annals of Physical and Rehabilitation Medicine (Association of spasticity and motor dysfunction in chronic stroke)

- Bhakta et al., 2000, J Neurol Neurosurg Psychiatry

- Brashear et al., 2002, New England Journal of Medicine

- Sun et al., 2010, Neurorehabilitation and Neural Repair

- Devier et al., 2017, Toxins

- Franck et al., 2021, NeuroRehabilitation

- Demetrios et al., 2014, Journal of Rehabilitation Medicine

The reviewer addresses an important point. Indeed, as the review focuses on the RS system we have moderated the aspect concerning 'spasticity':
The word 'beneficial' was deleted. The statement reads now

'Thus, the influence of the RS system on the development of spastic muscle tone can be important in so far that it enables some mobility, by supporting the body during stepping' (Dietz & Sinkjaer, 2007)'. (p 11)

Would this be ok for you?

In my mind (V. Dietz) this comment is based on a misunderstanding. I agree with the reviewer and most of the studies referenced dealing with clinical spasticity (e.g. effect of Botox injections on spastic muscle tone). I also agree that there are differences between upper and lower limb spasticity.
Spastic muscle tone, together with paresis, per se impairs mobility of upper and lower limbs. However, in our earlier studies we talk about spastic movement disorder that differs basically from clinical spasticity (outlined in the review Dietz and Sinkjaer, Lancet Neurol, 2007):
After a stroke or SCI, initially a flaccid paresis dominates and the patient becomes immobile (similar as following polyneuritis). In these cases, only the development of spastic muscle tone enables to regain some mobility. This is achieved on the basis of a simpler muscle tone regulation. Of course, spastic muscle tone can overshoot (severe SCI) and can lead to joint contractures (as following immobilization due to limb casting). This can be prevented by active and passive limb movements. With increasing recovery from the motor deficit less spastic muscle tone is required for the partial 'compensation' of paresis at a lower level of regulation (in the studies referenced, mobility was not a focus) Only in so far spastic muscle tone can be 'beneficial' and enables limited mobility.

Dear Professor Dietz,

Re: JP-TR-2026-290565R2 "**Impact of the brainstem in normal and impaired human movement control**" by Volker Dietz and Linard Filli

Thank you for submitting your manuscript to The Journal of Physiology. It has been assessed by a Reviewing Editor and by 0 expert referees and we are pleased to tell you that it is acceptable for publication following satisfactory revision.

Please share this decision with your co-authors as soon as possible.

The referee reports are copied at the end of this email.

Please address all the points raised and incorporate all requested revisions or explain in your Response to Referees why a change has not been made. We hope you will find the comments helpful and that you will be able to return your revised manuscript within 4 weeks. If you require longer than this, please contact journal staff: jp@physoc.org.

Your revised manuscript should be submitted online using the link in your Author Tasks: Link Not Available. This link is accessible via your account as Corresponding Author; it is not available to your co-authors. If this presents a problem, please contact journal staff (jp@physoc.org). Image files from the previous version are retained on the system. Please ensure you replace or remove any files that are being revised.

If you do not wish to submit a revised version of your manuscript, you must inform our journal staff (jp@physoc.org) or reply to this email to request withdrawal. Please note that a manuscript must be formally withdrawn from the peer review process at one journal before it may be submitted to another journal.

TRANSPARENT PEER REVIEW POLICY: To improve the transparency of its peer review process, The Journal of Physiology publishes online as supporting information the peer review history of all articles accepted for publication. Readers will have access to decision letters, including Editors' comments and referee reports, for each version of the manuscript, as well as any author responses to peer review comments. Referees can decide whether or not they wish to be named on the peer review history document.

ABSTRACT FIGURES: Authors may use The Journal's premium BioRender account to create/redraw their Abstract Figures (and any other suitable schematic figure). Information on how to access this account is here: https://physoc.onlinelibrary.wiley.com/journal/14697793/biorender-access.

This will enable Authors to create and download high-resolution figures. If authors have used the free BioRender service, they can use the instructions provided in the link above to download a high-resolution version suitable for publication. The link provided should only be used for the purposes of this submission. Authors will be charged for figures created on this account if they are not related to this manuscript submission.

LANGUAGE EDITING AND SUPPORT FOR PUBLICATION: If you would like help with English language editing, or other article preparation support, Wiley Editing Services offers expert help, including English Language Editing, as well as translation, manuscript formatting, and figure formatting at www.wileyauthors.com/eeo/preparation. You can also find resources for Preparing Your Article for general guidance about writing and preparing your manuscript at www.wileyauthors.com/eeo/prepresources.

REVISION CHECKLIST: Upload a full Response to Referees file. To create your 'Response to Referees' copy all the reports, including any comments from the Senior and Reviewing Editors, into a Microsoft Word, or similar, file and respond to each point, using font or background colour to distinguish comments and responses and upload as the required file type.

Please upload two versions of your manuscript text: one with all relevant changes highlighted and one clean version with no changes tracked. The manuscript file should include all tables and figure legends, but each figure/graph should be uploaded as separate, high-resolution files.

You may also upload:

- 'Potential Cover Art' for consideration as the issue's cover image
- Appropriate Supporting Information (Video, audio or data set: see https://jp.msubmit.net/cgi-bin/main.plex?form_type=display_requirements#supp).

We look forward to receiving your revised submission.

If you have any queries, please reply to this email and we will be pleased to advise.

Yours sincerely,

Laura Bennet
Senior Editor
The Journal of Physiology

EDITOR COMMENTS

Reviewing Editor:

The manuscript has improved, but several key points remain insufficiently addressed and require one final revision.

First, the discussion of responses to electrical and loud acoustic stimulation still overstates the evidence for a shared reticulospinal pathway. Similarities in latency, waveform, or muscle distribution are consistent with a common brainstem contribution, but they do not demonstrate that the responses are mediated by the same neurons or pathway. Please remove terms such as "identical," state clearly that the evidence is indirect, and acknowledge that cortical, subcortical, or spinal contributions cannot be excluded.

Second, the explanation of altered afferent responses after stroke still relies too strongly on impaired cortico-reticulospinal input. Because afferent signals can reach the reticular formation directly through brainstem sensory pathways, impaired cortico-reticular input should be presented only as a possible modulatory factor, and the proposed mechanism should be identified as speculative.

Third, the statements regarding spasticity remain too strong. It would be worth distinguishing a possible contribution of lower-limb extensor tone to weight support in selected patients from the broader functional effects of spasticity. Currently the manuscript still seems to imply that spasticity is generally necessary or beneficial.

A careful line-by-line language edit is also still required, as several grammatical and typographical errors remain.

-not only in the stimulated but also in of the contralateral limb(s)

-canditates

-stimulation evoke

-betweeen

-successionduring

-formation suggest

-oberserved

Senior Editor:

Thank you for your revisions, please take time to go through the manuscript thoroughly as the reviewer suggests.

END OF COMMENTS

Dear Editors,

Thank you for further comments on our manuscript which we have taken into account. We have revised the manuscript as requested by the Reviewing Editor.

EDITOR COMMENTS

Reviewing Editor:

The manuscript has improved, but several key points remain insufficiently addressed and require one final revision.

First, the discussion of responses to electrical and loud acoustic stimulation still overstates the evidence for a shared reticulospinal pathway. Similarities in latency, waveform, or muscle distribution are consistent with a common brainstem contribution, but they do not demonstrate that the responses are mediated by the same neurons or pathway. Please remove terms such as "identical," state clearly that the evidence is indirect, and acknowledge that cortical, subcortical, or spinal contributions cannot be excluded.

We reformulated the mentioned sections to interpret the similarity of responses to electrical and loud acoustic stimuli more carefully. As requested, we avoided too strong statements and mentioned that the evidence for a shared reticulospinal pathway is indirect.
The responses to unilateral nerve stimulation closely resemble on the two sides. It seems, therefore, rather unlikely that different neural structures contribute to their generation.
Nevertheless, we can hardly exclude any indirect cortical, subcortical or spinal influence on the neural coupling mechanism. We now mentioned this in the revised manuscript.

Second, the explanation of altered afferent responses after stroke still relies too strongly on impaired cortico-reticulospinal input. Because afferent signals can reach the reticular formation directly through brainstem sensory pathways, impaired cortico-reticular input should be presented only as a possible modulatory factor, and the proposed mechanism should be identified as speculative.

We adjusted the respective section and now highlight that the proposed mechanism relies on an indirect effect at the brainstem level that might be modulated by severed cortico-reticular projections. We further mention that the proposed explanation is speculative. Other neural mechanisms may contribute to the phenotype in stroke survivors.

Third, the statements regarding spasticity remain too strong. It would be worth distinguishing a possible contribution of lower-limb extensor tone to weight support in selected patients from the broader functional effects of spasticity. Currently the manuscript still seems to imply that spasticity is generally necessary or beneficial.

We have further weakened the statement regarding the role of spastic muscle tone in movement disorder.
I (VD) have, however, problems with the statement of the Reviewing Editor for two reasons:
First, we write a 'Topical Review' and refer to several earlier and recent publications in the field. Spastic muscle tone after e.g. a stroke or SCI was shown to be required in leg extensor muscles to support the body during stepping. A similar need for spastic muscle tone exists also in upper limbs after a cervical lesion. Flaccid paresis occurs in upper limbs after a cervical SCI, impeding the re-establishment of hand function, i.e. some spastic muscle tone is required to perform simple grasp movements (Dietz and Curt 2006). (See also my personal note to Reviewer 2, last revisions).
Second, why not to argue the other way around as the Reviewing Editor: Spastic muscle tone is basically beneficial to compensate for paresis (as argued in several papers). However, spastic muscle tone can overshoot (usually in severely affected, immobilized patients). In such cases therapeutic

measures are needed to reduce muscle tone. Some pathophysiological and clinical evidence supports this view.

A careful line-by-line language edit is also still required, as several grammatical and typographical errors remain.
Thank you for pointing out these grammatical and typographical errors. We have corrected them throughout the manuscript and carefully reviewed the entire text once again to identify and address any remaining issues.

Senior Editor:

Thank you for your revisions, please take time to go through the manuscript thoroughly as the reviewer suggests.

Dear Professor Dietz,

Re: JP-TR-2026-290565R3 "**Impact of the brainstem in normal and impaired human movement control**" by Volker Dietz and Linard Filli

We are pleased to tell you that your paper has been accepted for publication in The Journal of Physiology.

TRANSPARENT PEER REVIEW POLICY: To improve the transparency of its peer review process, The Journal of Physiology publishes online as supporting information the peer review history of all articles accepted for publication. Readers will have access to decision letters, including Editors' comments and referee reports, for each version of the manuscript, as well as any author responses to peer review comments. Referees can decide whether or not they wish to be named on the peer review history document.

The last Word (or similar) version of the manuscript provided will be used by the Production Editor to prepare your proof. When this is ready you will receive an email containing a link to Wiley's Online Proofing System. The proof should be thoroughly checked and corrected as promptly as possible.

Authors should note that it is too late at this point to offer corrections prior to proofing. Major corrections at proof stage, such as changes to figures, will be referred to the Editors for approval before they can be incorporated. Only minor changes, such as to style and consistency, should be made at proof stage. Changes that need to be made after proof stage will usually require a formal correction notice.

All queries at proof stage should be sent to: TJP@wiley.com.

Are you on Twitter? Once your paper is online, why not share your achievement with your followers? Please tag The Journal (@jphysiol) in any tweets and we will share your accepted paper with our 30,000 followers!

Yours sincerely,

Laura Bennet
Senior Editor
The Journal of Physiology

P.S. - You can help your research get the attention it deserves! Check out Wiley's free Promotion Guide for best-practice recommendations for promoting your work at www.wileyauthors.com/eeo/guide. You can learn more about Wiley Editing Services which offers professional video, design, and writing services to create shareable video abstracts, infographics, conference posters, lay summaries, and research news stories for your research at www.wileyauthors.com/eeo/promotion.

• IMPORTANT NOTICE ABOUT OPEN ACCESS: To assist authors whose funding agencies mandate immediate public access to published research findings, The Journal of Physiology allows authors to pay an Open Access (OA) fee to have their papers made freely available immediately on publication.

The Corresponding Author will receive an email from Wiley with details on how to register or log in to Wiley Authors where you will be able to place an order.

You can check if your funder or institution has a Wiley Open Access Account here:
https://authors.wiley.com/author-resources/Journal-Authors/open-access/author-compliance-tool.html

MEMBER DISCOUNT: Corresponding authors who are Members of the Physiological Society are eligible for a 20% discount on the cost of the Article Processing Charge (APC). To claim this discount, the Corresponding Author will need to log in to the Physiological Society Membership portal and retrieve the discount code (https://www.physoc.org/journals-and-media/journals/journal-discount-for-members/). This code should then be quoted in the billing section of Wiley Authors when selecting the open access option.

EDITOR COMMENTS

Reviewing Editor:

Thank you for your careful revision of the manuscript and your detailed responses to the reviewers' comments. In my view, the remaining concerns have now been adequately addressed.
